# Construction of an aerolysin-based multi-epitope vaccine against *Aeromonas hydrophila:* an *in silico* machine learning and artificial intelligence-supported approach

**DOI:** 10.3389/fimmu.2024.1369890

**Published:** 2024-03-01

**Authors:** Abdullah S. Alawam, Maher S. Alwethaynani

**Affiliations:** ^1^ Department of Biology, College of Science, Imam Mohammad Ibn Saud Islamic University (IMSIU), Riyadh, Saudi Arabia; ^2^ Department of Clinical Laboratory Sciences, College of Applied Medical Sciences, Shaqra University, Al-Quwayiyah, Saudi Arabia

**Keywords:** immunoinformatics, epitopes, MD simulations, systems biology, vaccine, *A. hydrophila*

## Abstract

*Aeromonas hydrophila*, a gram-negative coccobacillus bacterium, can cause various infections in humans, including septic arthritis, diarrhea (traveler’s diarrhea), gastroenteritis, skin and wound infections, meningitis, fulminating septicemia, enterocolitis, peritonitis, and endocarditis. It frequently occurs in aquatic environments and readily contacts humans, leading to high infection rates. This bacterium has exhibited resistance to numerous commercial antibiotics, and no vaccine has yet been developed. Aiming to combat the alarmingly high infection rate, this study utilizes *in silico* techniques to design a multi-epitope vaccine (MEV) candidate against this bacterium based on its aerolysin toxin, which is the most toxic and highly conserved virulence factor among the *Aeromonas* species. After retrieval, aerolysin was processed for B-cell and T-cell epitope mapping. Once filtered for toxicity, antigenicity, allergenicity, and solubility, the chosen epitopes were combined with an adjuvant and specific linkers to create a vaccine construct. These linkers and the adjuvant enhance the MEV’s ability to elicit robust immune responses. Analyses of the predicted and improved vaccine structure revealed that 75.5%, 19.8%, and 1.3% of its amino acids occupy the most favored, additional allowed, and generously allowed regions, respectively, while its ERRAT score reached nearly 70%. Docking simulations showed the MEV exhibiting the highest interaction and binding energies (−1,023.4 kcal/mol, −923.2 kcal/mol, and −988.3 kcal/mol) with TLR-4, MHC-I, and MHC-II receptors. Further molecular dynamics simulations demonstrated the docked complexes’ remarkable stability and maximum interactions, i.e., uniform RMSD, fluctuated RMSF, and lowest binding net energy. *In silico* models also predict the vaccine will stimulate a variety of immunological pathways following administration. These analyses suggest the vaccine’s efficacy in inducing robust immune responses against *A. hydrophila*. With high solubility and no predicted allergic responses or toxicity, it appears safe for administration in both healthy and *A. hydrophila*-infected individuals.

## Introduction

*Aeromonas hydrophila* (*A. hydrophila*) is a gram-negative, motile, non-sporulating, coccobacillus or rod-shaped, oxidase-positive, H2S-positive, indole-positive, facultative anaerobe bacteria that belongs to the family *Aeromonadaceae* (1). On blood agar, it results in beta-hemolysis and has the ability to ferment carbohydrates, producing gas and acid (2). While its presence in soil has also been observed, it is primarily found in the aquatic environment, both fresh and marine waters (3). *A. hydrophila* is an inhabitant of fish, amphibians, and reptiles (4). Fish in rivers, estuaries, and saltwater are the main reservoirs (2). Additionally, it has proven possible to separate it from chlorinated water sources. The list of potential contaminants for developing water-borne diseases maintained by the United States Environmental Protection Agency includes *Aeromonas* species (5).

*A. hydrophila* species, which initially emerged from human feces in 1937 (3), not only infects aquatic creatures but is also linked to a variety of infectious disorders that affect both infants and adults. Geographically, infections with *A. hydrophila* happen everywhere, and it has been reported in more than 20 countries on six continents (6). The majority of *A. hydrophila* infections occur in tropical and semitropical countries, with only a small number of occurrences in temperate locations (7). As mentioned, fishes are the main reservoirs, and when these fishes are eaten by locals, it can cause a variety of infections in them. This bacterium releases a number of toxins into the water that is readily available to humans in the form of drinking water and seafood, including aerolysin, hemolysin, proteases, lipases, lecithinases, amylases, and DNases (1). The infections include septic arthritis, diarrhea (traveler’s diarrhea), gastroenteritis, skin and wound infections, meningitis, fulminating septicemia, enterocolitis, peritonitis, endocarditis, urinary tract infections (UTIs), hematologic malignancy, hepatic cirrhosis, ocular infection, pneumonia, tonsillitis, endocarditis, osteomyelitis, epiglottitis, liver abscess, pleural empyema, cholangitis, thrombophlebitis, and hemolytic uremic syndrome (HUS) (6). The incubation period of this bacterium is 1–2 days, and mortality among the population is 25%–30% (8).

*A. hydrophila* virulence factors are what determine its pathogenicity. Aerolysin is the most important toxin that contributes significantly to a range of infections in people infected by *A. hydrophila* (9). When furin protease activates aerolysin, which is produced as an inactive precursor, the toxin diffuses toward the cell and creates a homo-heptameric pore on the target cells, which can cause cell death (10). Pores formed by aerolysin cause osmotic imbalances in target cells, G-protein activation, and cell lysis (11). This toxin consists of two subunits and is composed of 493 amino acids, and strains lacking the aerolysin-encoding gene showed a significant reduction in infectivity (12). Epithelial cells are mostly vulnerable to this toxin, but studies show that erythrocytes, fibroblast cells, lymphocytes, and granulocytes are also easily destroyed (13).

Antibiotic misuse fuels the fire of bacterial resistance, of which *Aeromonas hydrophila* is a prime example. This globally pervasive bacterium has developed multi-resistance to a formidable arsenal of antibiotics, including penicillins, cephalosporins, lincosamides, and nalidixic acid. Particularly alarming is the high prevalence of extended-spectrum cephalosporin resistance in *A. hydrophila*, jeopardizing a critical line of defense against severe infections (14).

It is well known that the use of vaccines has saved millions of people against many fatal infections in the past by boosting their immune systems against those causative pathogens (15). Keeping in view the increasing ratio of *A. hydrophila* infections and the increased rate of antibiotic resistance across the community, vaccine production should be considered a primary and important step to overcome this problem. Traditional vaccine production takes years to synthesize an effective vaccine and is much more expensive, which can lead to late and less availability to the population, resulting in increased infection and resistance rates (16).

Modern techniques are being introduced to synthesize effective vaccine candidates in less time using bioinformatics approaches (17). Recently, the deadly pandemic of coronavirus was successfully controlled by the introduction of a vaccine against it in less than a year using bioinformatics approaches (18). The use of essential microbial proteins to map epitopes complementary to human immune receptors is known as “*in silico* vaccine designing”, which is a fairly novel technique (19). Some epitopes have the ability to stimulate the immune system when they are introduced into the body, which will help fight off the associated microbes (18). To date, thousands of vaccine candidates are designed against many deadly pathogens using these techniques (20). These methods are cost-effective and have reduced production time to a great extent. Keeping this importance in mind, our study focuses on the production of a potential vaccine candidate against *A. hydrophila* using bioinformatics approaches so that the community could be saved from this fatal pathogen.

This study was initiated by designing a multi-epitope vaccine (MEV) against the aerolysin toxin of *A. hydrophila.* As mentioned, aerolysin is the most potent virulence factor of *A. hydrophila*, and it is produced in most infections (10). Studies have also suggested that strains lacking the genes responsible for aerolysin production are noninfectious and nonpathogenic (21). Keeping in mind the importance of aerolysin and its destructive effects, designing a MEV against it could reduce the infection rate in the community by evoking the immune system in the form of cell-mediated and humoral immunity.

MEV development starts by retrieving the data of aerolysin toxin in the form of amino acid sequence and subjecting it to B-cell epitope prediction. The obtained epitopes were processed for MHC-II and MHC-I epitope prediction, and the resulting epitopes were processed for essentiality analysis using different bioinformatics tools. Furthermore, the structure was predicted for the vaccine candidate, and its binding efficiency was checked with the major immune receptors of humans, i.e., MHC-I, MHC-II, and TLR4, and the docked complexes were processed for simulation. At last, the vaccine was produced via *in silico* cloning using the SnapGene tool. Our work shows accepting results that this vaccine will work efficiently and will suppress the onset and progression of *A. hydrophila* infection in healthy and infected individuals.

## Research workflow

**Figure 1:**
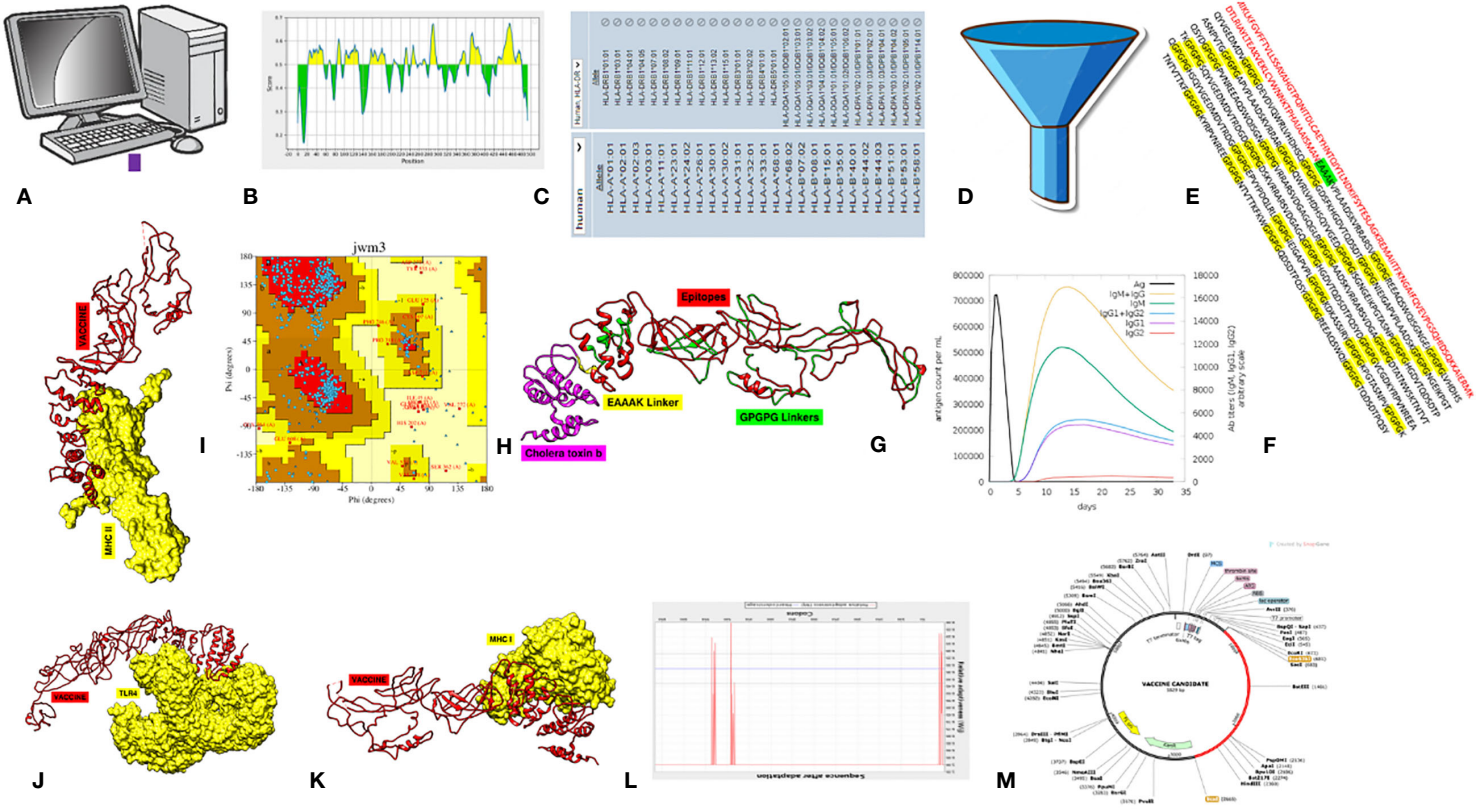
Schematic methodology flow of the study. **(A)** Data retrieval. **(B)** B-cell epitope prediction. **(C)** T-cell epitope prediction. **(D)** Epitope essentiality filtration. **(E)** Final MEV construct. **(F)** Immune simulation. **(G)** Structure prediction. **(H)** Stability analysis. **(I–K)** Molecular docking with MHC-II, TLR4, and MHC-II and simulations. **(L)** Jcat analysis. **(M)** *In silico* cloning.

To secure our aim, a consistent, step-by-step process was employed, as shown in **Figure 1**.

### Data mining, retrieval, homology, and conservancy check

The study starts by selecting aerolysin as the potential candidate to synthesize a MEV against it. The virulence of aerolysin was confirmed via an online tool called the Virulence Factor Database (VFDB) (22). This database consists of thousands of virulence factors associated with different known pathogens. Furthermore, clinical case studies and the role of aerolysin in causing different infections in humans were confirmed by PubMed and UniProt (23). The amino acid sequence of aerolysin was obtained from the UniProt database, and the structure was obtained from the Protein Database (PDB) with ID (1PRE). Obtained sequences were processed for homology checks against *Homo sapiens*, *Lactobacillus rhamnosus*, *Lactobacillus casei*, and *Lactobacillus johnsonni* by using BLASTp from the NCBI server (24). This was done to check that the aerolysin sequence does not have any similarity with the human and normal flora proteome. If similarity was shown, the study would not proceed because the obtained epitopes may provoke autoimmunity (25). Additionally, a conservancy analysis was conducted on aerolysin using the Blastp tool (24) to assess the occurrence of this virulence factor in other strains of the *Aeromonas* family. The findings from this analysis will enhance the significance of aerolysin-based MEV if the majority of strains harbor this gene in their genome. Consequently, such a MEV could confer broad immunity against all strains of *Aeromonas* that possess aerolysin.

### Immune Epitope Prediction analysis

The obtained sequence of aerolysin was subjected to epitope prediction. The Immune Epitope Prediction (IEDB) database is a unique platform that consists of millions of known and predicted epitopes related to critical immune cell receptors, i.e., TLR4, BCR, MHC-II, MHC-I, etc. (26, 27). The system operates on machine learning algorithms to predict epitopes from the query sequence, leveraging the chosen alleles as a basis. This methodology allows for the accurate identification of epitopes tailored to specific alleles, enhancing the precision and effectiveness of the MEV candidate. The MEV designed from the predicted epitopes has demonstrated success across multiple vaccine design projects when tested experimentally (28–30). This validation underscores the efficacy and versatility of the MEV approach, further supporting its potential as a promising solution in vaccine development (31). Our study focuses on B-cell epitope prediction, and the obtained epitopes were separately processed for MHC-I and MHC-II epitope prediction. For the prediction of MHC-II and MHC-I epitopes, all known alleles representing the entire human population were selected. This approach ensures that the predicted epitopes encompass a broad spectrum of alleles related to the immune system, thereby enhancing the versatility and applicability of the MEV for experimental approaches (32). The resulting epitopes were selected on the basis of low percentile ranking because a lower ranking score resulted in the maximum binding of the epitopes to immune receptors (33, 34).

### Antigenicity, toxicity, solubility, and allergenicity analyses of the predicted epitopes

The obtained epitopes were subjected to a series of analyses so that filtered epitopes could be obtained and used as part of MEV. Antigenicity and toxicity were checked for each epitope using Vexigen 2.0 (35) and the ToxinPred tool (36). Epitopes with no toxicity and an antigenicity value of less than or equal to 0.4 underwent additional solubility analysis. Each epitope’s solubility was assessed using the Innovagen Peptide Calculator, and those with good water solubility were processed further (37). To check for allergic reactions, filtered epitopes were examined using the Allertop 2.0 tool. Only those epitopes that were negative for allergenicity were further processed (38).

### Finalizing vaccine candidate sequence and its immune simulation

The vaccine construct was assembled by sequentially linking the filtered epitopes. An adjuvant was initially conjugated to the first epitope via an EAAAK linker. Subsequently, GPGPG linkers were utilized to connect each subsequent epitope in a tandem array (39). The purpose of adding an adjuvant was to enable the MEV to elicit a strong immunological response, and the objectives of adding linkers were to stabilize the vaccine and prevent self-complementary binding of its sequences (40). The resulting construct’s physiochemical properties were also anticipated to verify its theoretical PI, solubility in water, stability, and other properties. Immune simulation tests were conducted for the vaccine construct using the c-IMMSIMM tool in order to evaluate immune responses (cellular and humoral) against the vaccine candidate (41).

### MEV structure prediction, refinement, and stability analysis

A three-dimensional structure was predicted for the MEV via an online structure modeling tool known as iTESSOR (42). iTESSOR uses a hierarchical approach that combines threading, structural refinement, and template-based fragment assembly (43). It uses particular methods to further enhance the structure after making a forecast. Because of this combined method, iTESSOR is now at the forefront of protein structure prediction, allowing for reliable structure prediction for a wide range of protein folds and sequences (44). The obtained model was further processed for refinement via the GALAXY refine tool in order to minimize steric clashes among the amino acids and increase the stability of the MEV by transforming coils into suitable helix structures (45). The final stability of the MEV was checked via an online tool known as ERRAT (46) and PDBsum, where a Ramachandran plot was obtained for the predicted MEV (47, 48).

### Molecular docking, interaction analysis, and simulations

Molecular docking was used to evaluate the MEV’s ability to bind to immune cell receptors. Using the ClusPro server, the vaccine construct was docked with MHC-I (PDB ID: 1I1Y), MHC-II (PDB ID: 1KG0), and TLR4 (PDB ID: 4G8A) receptors (49). The complexes were further visualized via the UCSF Chimera tool (50), and the PDBsum tool was used to analyze the kinds and quantities of interactions among the docked complexes (51). Furthermore, the docked complexes were processed through molecular dynamic simulation (MDS), which is a method employed to comprehend molecular behavior and characteristics at the atomic level. It proves effective in assessing the binding stability of a complex, such as a ligand protein, within a dynamic environment. The AMBER 20 package was used, in which the system underwent 500 steps of steepest descent and 500 stages of conjugate gradient minimization (52). Position constraints with a force constant of kcal mol^−1^ Å^−2^ were used to keep the protein stable. Subsequently, there were 2,000 more steepest descent stages and 2,000 more conjugate gradient minimization phases, respectively. Secondly, the system was heated to the target temperature of 300 K for 20 ps under weak positional constraints on the protein atoms (force constant of 10 kcal mol^−1^ Å^−2^) and constant volume periodic boundary conditions (NVT). After that, the system was calibrated by running a production simulation for 100 ns while maintaining the same pressure and temperature (NPT) for roughly 40 ns. Using isotropic position scaling and a relaxation duration of 2 ps, an average pressure of 1 atm was maintained. Langevin dynamics with a collision frequency of one ps-1 were used to control the temperature (53). The Particle Mesh Ewald (PME) method was utilized to handle nonbonded interactions and long-range electrostatic interactions, with a 10-Å limit (54). The numerical integration time step was 2 fs, and the SHAKE method was utilized to limit all bonds containing hydrogen (55). VMD and the PTRAJ program from the Amber11 package were used to analyze the simulation results (56). For receptor–binder complex systems, the binding free energy (ΔGbinding) was calculated using molecular mechanics with a generalized Born and surface area solvation (MM/GBSA) approach (57). Throughout the simulation trajectory, 1,000 pictures were captured at 20 ns intervals in order to calculate the MM/GBSA free energy difference.

### Codon optimization and *in silico* cloning

The designed MEV showing all the important properties of a common vaccine was subjected to *in silico* cloning (58). This was achieved by performing codon optimization of the MEV via an online tool called Jcat (59). Jcat tool sets the sequence of the MEV according to the codon usage of the expression system used in *in silico* cloning (*Escherichia coli K-12* was used as an expression system). The processed sequence was inserted into a well-known vector named pet28+(a) via the SnapGene tool (60). Due to its T7 promoter, several cloning sites, His-tag fusion (polyhistidine that makes it easier to purify the produced protein), and a selectable marker, this vector is primarily utilized in cloning techniques (61). This process will yield accurate data that experimentalists may use to allow manufacturing at the industrial level.

## Results

### Data retrieval

The VFDB showed that aerolysin is among the most essential virulence factors of *A. hydrophila* and plays important roles in human-related infections. The sequence was obtained from the UniProt database, and the structural ID for aerolysin was obtained from PDB, as shown in **Table 1**.

**Table 1:** The amino acid sequence of aerolysin, along with its PDB ID and residue number.

Virulence factor/PDB ID/NCBI ID/residues	Sequence
**Aerolysin OS=*Aeromonas hydrophila*/WP_098980947.1/1PRE/493**	MQKIKLTGLSLIISGLLMAQAQAAEPVYPDQLRLFSLGQGVCGDKYRPVNREEAQSVKSN IVGMMGQWQISGLANGWVIMGPGYNGEIKPGTASNTWCYPTNPVTGEIPTLSALDIPDGD EVDVQWRLVHDSANFIKPTSYLAHYLGYAWVGGNHSQYVGEDMDVTRDGDGWVIRGNNDG GCDGYRCGDKTAIKVSNFAYNLDPDSFKHGDVTQSDRQLVKTVVGWAVNDSDTPQSGYDV TLRYDTATNWSKTNTYGLSEKVTTKNKFKWPLVGETELSIEIAANQSWASQNGGSTTTSL SQSVRPTVPARSKIPVKIELYKADISYPYEFKADVSYDLTLSGFLRWGGNAWYTHPDNRP NWNHTFVIGPYKDKASSIRYQWDKRYIPGEVKWWDWNWTIQQNGLSTMQNNLARVLRPVR AGITGDFSAESQFAGNIEIGAPVPLAADSKVRRARSVDGAGQGLRLEIPLDAQELSGLGF NNVSLSVTPAANQ

### Homology and conservancy check

Each virulence factor was separately aligned via BLASTp tool against the human proteome and important normal flora bacteria, i.e., *Lactobacillus rhamnosus*, *Lactobacillus casei*, and *Lactobacillus johnsonni*, in order to check if there is sequence similarity or not. The sequence of each organism showed no such similarity with the virulence factor, and it was safe to use for further analysis. This was done because, while constructing such a vaccine candidate, one should keep in mind that the sequence used in constructing MEV should not resemble the sequence of these organisms because it may lead to autoimmune disorders or abnormal killing of the normal flora of the human body because our adaptive immunity can be provoked against our own body and normal flora. Secondly, sequence alignment of aerolysin with all known strains of the *Aeromonas* family reveals that the selected virulence factor is present in all those strains of *Aeromonas* known for causing serious infections in the human population. **Supplementary Table S1** shows that aerolysin is present in 17 strains of *A. hydrophila*, 39 strains of *Aeromonas* spp., three strains of *A. salmonicida*, and each strain of *A. bestiarum*, *A. piscicola*, *A. caviae*, and *A. dhakensis*, respectively. Alignment scores of the aerolysin with the mentioned strains showed a maximum result of 95%–100%, which clearly illustrates that these genes are exactly present in these mentioned strains.

### B-cell epitope prediction phase

**Figure 2:**
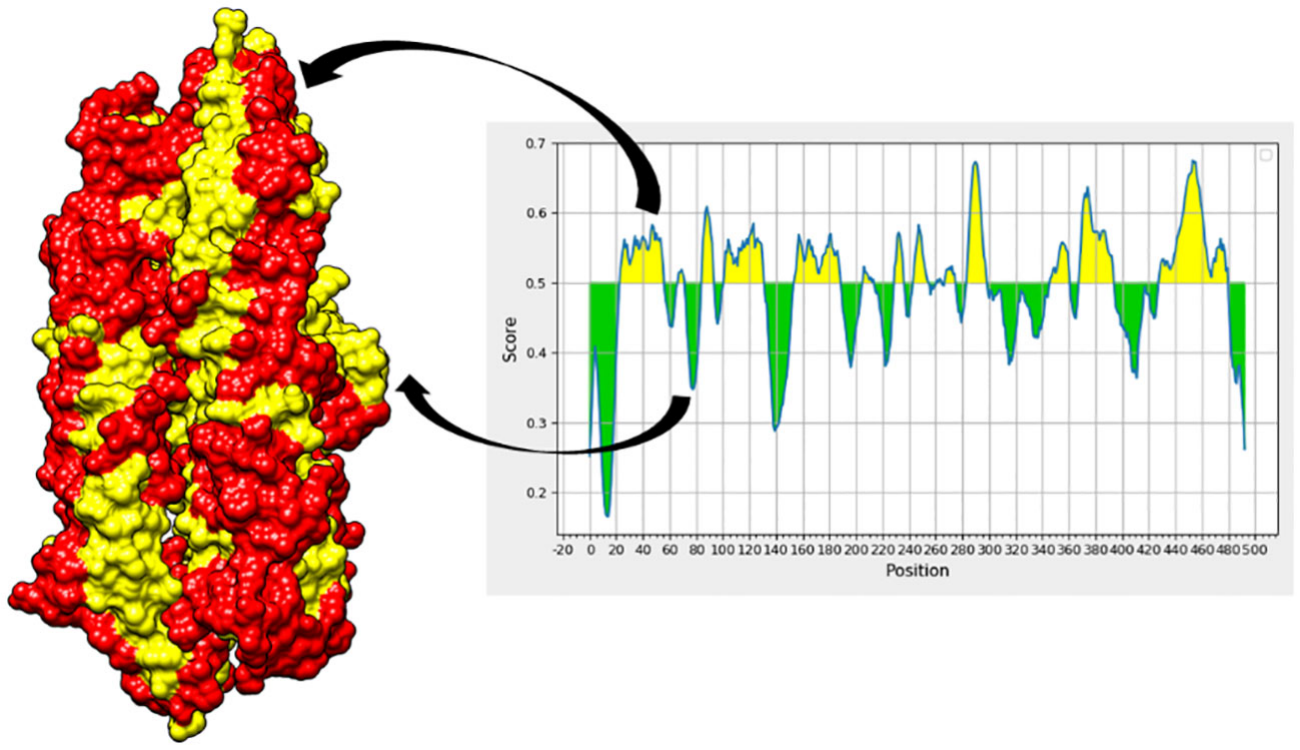
In the aerolysin structure, the red regions showed B-cell epitopes (represented by the yellow region of the graph), while the yellow regions in the structure represent amino acids that do not contribute to the mentioned epitopes and are represented by the green regions of the graph, respectively.

The chosen proteins were ranked in order of priority for immunological epitope prediction. This was accomplished by first predicting the B-cell epitope and then the T-cell epitope. To find T-cell epitopes, additional processing was done on the B-cell epitopes. B cells, macrophages, and cytotoxic T lymphocytes are all stimulated by helper T lymphocytes. On the other hand, antigens can be directly recognized by cytotoxic T cells (62). Conversely, B cells can develop into plasma cells, which produce antibodies (63). Aerolysin was subjected to B-cell epitope prediction. The sequence was filtered and spliced into 14 linearly predicted B-cell epitopes shown in **Table 2**. All the epitopes were also presented schematically in **Figure 2**, which showed that epitopes present in the yellow region above the threshold value of 0.5 are considered B-cell epitopes, while sequences below the threshold present in the green region do not belong to the B-cell epitope category (26). Out of 493 amino acids of aerolysin, 262 amino acids (selected from each chain, i.e., A and B of aerolysin) were further processed separately for MHC-II and MHC-I epitope prediction. All 262 epitopes of B cells were used for T-cell epitope prediction. The first MHC-II epitopes were predicted, followed by MHC-I epitope prediction. For MHC-II epitope prediction, the IEDB-recommended method was used for epitope prediction (64), and all the known alleles of MHC-II molecules were selected. This was done because the selection of more alleles results in diverse epitopes that could be present in a high percentage of people. Epitopes with a percentile score of ≤ 10.0 were selected, and the remaining were rejected because a lower percentile score results in more efficient binding. For MHC-I epitope prediction, the same IEDB-recommended method was used (64), and all the known alleles for MHC-I were selected. Two filtration thresholds were set for choosing the best epitopes, i.e., top 1% epitopes and epitopes having a percentile score of ≤ 1.0 were selected. Top 1% was selected because this would filter those epitopes that are common in all the selected alleles, and the lowest percentile score results in better binding with the immune receptors. The obtained epitopes of MHC-II and MHC-I are shown in **Table 3**.

**Table 2:** The table shows detailed information about the B-cell epitopes, i.e., their location in the protein structure plus their length and residues.

Protein	Protein length	Epitope location (amino acid positions)	Epitope length (number of amino acids)
Aerolysin	493aa	24–56; 68–72; 85–94; 102–131; 155–189; 206–214; 230–236; 244–255; 262–265; 268–275; 284–299; 347–361; 369–394; 429–480	33aa; 5aa; 10aa; 30aa; 35aa; 9aa; 7aa; 12aa; 4aa; 8aa; 16aa; 15aa; 26aa; 52aa **Total = 262**
**Residues detail w.r.t epitope length**			
AEPVYPDQLRLFSLGQGVCGDKYRPVNREEAQS			
WQISG			
NGEIKPGTAS			
NPVTGEIPTLSALDIPDGDEVDVQWRLVHD			
HSQYVGEDMDVTRDGDGWVIRGNNDGGCDGYRCGD			
SFKHGDVTQ			
DSDTPQS			
YDTATNWSKTNT			
VTTK			
FKWPLVGE			
ANQSWASQNGGSTTTS			
WGGNAWYTHPDNRPN			
GPYKDKASSIRYQWDKRYIPGEVKWW			
AESQFAGNIEIGAPVPLAADSKVRRARSVDGAGQGLRLEIPLDAQELSGLGF			

**Table 3:** Obtained epitopes for MHC-II (15 mers) and MHC-I (9–10 mers) along with their percentile scores.

MHC-II epitopes	Percentile score	MHC-I epitopes	Percentile score
VPLAADSKVRRARSV	0.2	EPVYPDQLRL	0.18
REEAQSWQISGNGEI	3.6	IEIGAPVPL	0.23
LVHDHSQYVGEDMDV	4.5	KDKASSIRY	0.4
DEVDVQWRLVHDHSQ	2.7	KPGTASNPV	0.19
GDSFKHGDVTQDSDT	1.3	KTNTVTTKF	0.01
NIEIGAPVPLAADSK	6.5	KYRPVNREE	0.11
NGEIKPGTASNPVTG	2.6	NTVTTKFKW	0.01
APVPLAADSKVRRAR	0.01	QDSDTPQSY	0.03
QWRLVHDHSQYVGED	0.11	REEAQSWQI	0.1
ISGNGEIKPGTASNP	5.0	TQDSDTPQSY	0.03
HGDVTQDSDTPQSYD	2.7		
PVNREEAQSWQISGN	4.0		
VRRARSVDGAGQGLR	8.3		
AADSKVRRARSVDGA	8.8		
DTATNWSKTNTVTTK	9.5		
SQYVGEDMDVTRDGD	3.8		
DSKVRRARSVDGAGQ	2.5		
HGDVTQDSDTPQSYD	1.8		
VCGDKYRPVNREEAQ	0.01		
HSQYVGEDMDVTRDG	1.4		

### Epitope filtration phase

Out of a multitude of possibilities, only the most promising epitopes made the cut for our vaccine design. After undergoing rigorous assessments for toxicity, solubility, allergenicity, and antigenicity, only a select few emerged victorious. **Table 4** showcases these epitopes, ready to serve as the building blocks of our MEV.

**Table 4:** Filtered epitopes for vaccine construct.

Selected epitopes	Antigenicity	Solubility	Allergenicity	Toxicity
VPLAADSKVRRARSV	**Antigen**	**Water soluble**	**Nonallergen**	**Nontoxin**
REEAQSWQISGNGEI				
LVHDHSQYVGEDMDV				
DEVDVQWRLVHDHSQ				
GDSFKHGDVTQDSDT				
NIEIGAPVPLAADSK				
NGEIKPGTASNPVTG				
APVPLAADSKVRRAR				
QWRLVHDHSQYVGED				
ISGNGEIKPGTASNP				
HGDVTQDSDTPQSYD				
PVNREEAQSWQISGN				
VRRARSVDGAGQGLR				
AADSKVRRARSVDGA				
DTATNWSKTNTVTTK				
SQYVGEDMDVTRDGD				
DSKVRRARSVDGAGQ				
HGDVTQDSDTPQSYD				
VCGDKYRPVNREEAQ				
HSQYVGEDMDVTRDG				
TQDSDTPQSY				
EPVYPDQLRL				
IEIGAPVPL				
KDKASSIRY				
KPGTASNPV				
KTNTVTTKF				
KYRPVNREE				
NTVTTKFKW				
QDSDTPQSY				
REEAQSWQI				

### Vaccine construction phase

**Figure 3:**
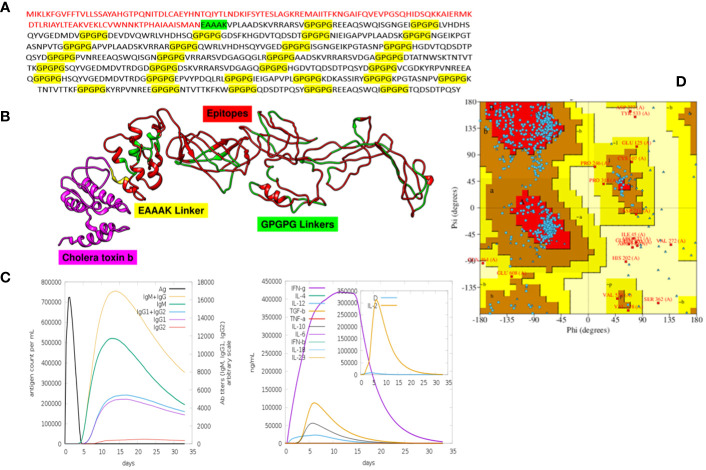
All these figures include crucial information about the MEV. **(A)** It is the final sequence of the MEV, where the adjuvant is highlighted in red, EAAAK in green, and GPGPG in yellow, and the nonhighlighted regions are the epitopes. **(B)** The three-dimensional structure of MEV. **(C)** The graphical visualization of immune responses against MEV. **(D)** The Ramachandran plot (for stability analysis) for the MEV.

In total, 30 unique epitopes were chosen from the list of combined epitopes after the aforementioned analyses. One of the main problems was resolved by creating a multi-epitope-based vaccination construct by joining different kinds of designated epitopes with certain GPGPG linkers. Furthermore, the EAAAK linker was used to link the epitope peptide and the adjuvant for the cholera toxin B component. Because GPGPG linkers can effectively block junctional folding and initiate an immunological response involving T-helper cells, they were placed between epitopes (65). EAAAK is a stiff, stable α-helical peptide linker with an intramolecular hydrogen bond and a closed-packed backbone. Consequently, in a fusion protein, the EAAAK linker serves as a domain spacer (66). Additionally, linkers facilitate the union of epitopes to form a significant structure with a polytope shape (67). Cholera toxin B was used as an adjuvant because it significantly increased the synthesis of IgA in the mucosa and other immune responses (68). The reason behind this is that it is nontoxic and has the ability to attach itself to the monosialotetrahexosylganglioside (GM1) receptor. This receptor can be found in the cytosols and on the membranes of various cells, such as B cells, macrophages, dendritic cells, gut epithelial cells, and antigen-presenting cells (69). In a similar vein, the adjuvant employed is risk-free and produces strong immune responses that are particular to the antigen with which it is coupled (70). **Figures 3A, B** and **Table 5** present the MEV construct and its significant attributes, respectively.

**Table 5:** Essential characteristics of the vaccination and its half-life within the human body.

	Molecular weight	No. of amino acids	Instability Index	Theoretical PI	GRAVY score	Antigenicity	Half-life
**Vaccine construct**	68,266.77	666	31.79	5.60	−0.815	Antigen	>20 h

### Immune simulations

The immunological reactions to the MEV were analyzed using the C-ImmSim server to ascertain if the epitopes would be sufficient to produce immunity (41). By employing this technique, it is also possible to determine the emergence of immunological interactions between the epitopes and specific targets. The ability of the MEV construct to elicit potent cellular and humoral immune responses is shown in **Figure 3C**. An increase in the development of adaptive responses, such as IgG and IgM antibodies, was seen in a C-immune simulation analysis carried out 35 days after the human immune system was virtually exposed to the highest dosage of vaccine antigen. Similarly, robust cellular immune responses were evident by significant production of interferon-gamma, interleukin (IL)-10, and IL-2 observed within 5 days postadministration. These findings highlight the MEV’s ability to stimulate potent immune responses, indicating its potential as an effective vaccine candidate (71–73).

### Vaccine structure modeling, refinement, and stability analysis

The MEV construct was modeled for structure prediction. In order to do this, the final MEV construct’s amino acid was uploaded in FASTA format to the iTESSOR program, and an *ab initio* modeling technique was used to predict the structure (42). Five different structures were forecast in total, and the one with the highest confidence score was chosen. The modeled structure was refined by reducing steric clashes among the residues, making it stable and suitable for further analysis (46, 74, 75). The stability of the MEV structure was predicted, and it showed acceptable values of stability. Two algorithms were used to predict the stability, i.e., the Ramachandran plot and the ERRAT plot. The Ramachandran plot illustrates the division and placement of the amino acids in the MEV construct into distinct areas, each of which denotes a distinct stability level (76). The four zones in this plot are the most favored areas (red), the additional permitted areas (brown), the generously allowed regions (yellow), and the disallowed regions (pale). Following the placement of the MEV construct residues in these areas, the combined stability was computed. Based on each residue’s phi and psi angles, which are shown on the plot’s *x*-axis and *y*-axis, residues are arranged in these regions. The majority of the MEV construct’s residues were found to be in the allowed region (75.5%), which was followed by the generously allowed zone (1.3%), the additional allowed regions (19.8%), and the disallowed region (2.4%) (**Table 6**). Our MEV construct is stable, as evidenced by the presence of more residues in the permitted regions and fewer in the prohibited zone (**Figure 3D**). The ERRAT score for structure stability also lies in the acceptable range of 70%, respectively.

**Table 6:** Statistical data from the Ramachandran plot.

	Vaccine	
	No. of residues	Percentage
Most favored regions (A, B, C)	325	75.5%
Additional allowed regions (a, b, l, p)	91	19.8%
Generously allowed regions (~a, ~b, ~1, ~p)	6	1.3%
Disallowed regions (XX)	11	2.4%
Non-glycine and nonproline residues	460	100%
End-residues (excl. Gly and pro)	2	
Glycine residues	121	
Proline residues	83	
Total number of residues	666	

### Molecular docking and interaction analysis

**Figure 4:**
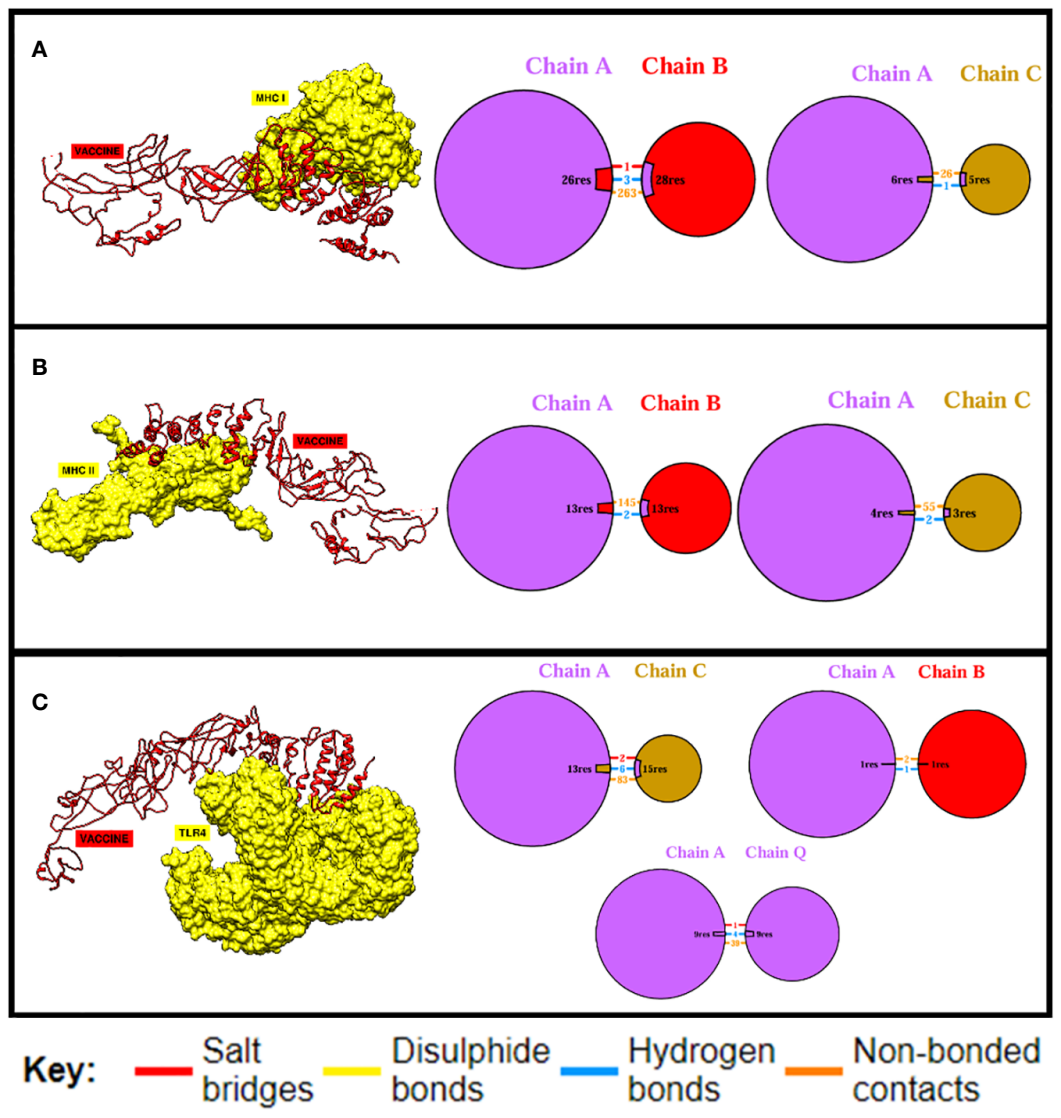
Binding analysis. The quantity and kind of interactions between the MEV construct’s chains and MHC-I chains are displayed in **(A)**. MHC-I and the MEV construct are shown to be bound and interacting, respectively, in **(B)**, while **(C)** displays the quantity and kind of interactions between TLR4 and the MEV construct.

Docking is an essential method for assessing how well two molecules bind together. The MEV candidate was bound to the most important receptors of the human immune system, i.e., MHC-I, MHC-II, and TLR4 in order to predict its binding efficiency. The pdb structures of the immune receptors were retrieved from the Protein Data Bank (PDB) (77) and were separately docked with the MEV, and binding free energies were calculated where the obtained values clearly demonstrate maximum binding (**Table 7**). Furthermore, the PDBsum tool was used to verify the kinds and quantities of interactions. The MEV exhibited effective binding with TLR4, MHC-I, and MHC-II, with the highest number of contacts. In addition, hydrogen bonds were observed among the complexes, providing additional evidence of maximal binds. **Figure 4** displays the structure of docked complexes as well as the quantity, kind, and number of interacting residues.

**Table 7:** The top 1 clusters of the docked complexes are displayed, together with the number of members engaged in the interaction and the binding energies of the complexes.

Docked complex	Cluster	Members	Binding energy
MHC I—VACCINE	TOP 1	84	−923.2 kcal/mol
MHC II–VACCINE	TOP 1	110	−988.3 kcal/mol
TLR 4–VACCINE	TOP 1	28	−1,023.4 kcal/mol

### *In silico* cloning

**Figure 5:**
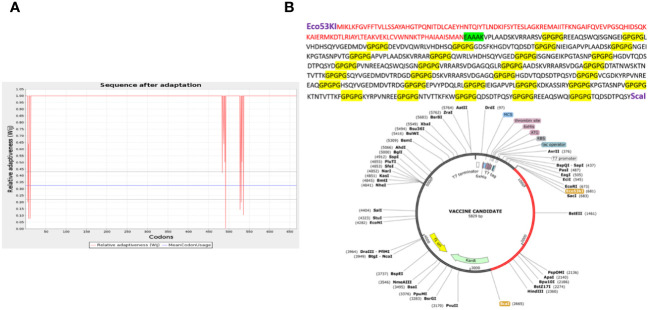
This graph shows that only a few codons (deviated red projections) cannot be adapted according to the expression system (increased red lines below the threshold 1.00), while the uniform red line along point 1.00 shows that the maximum of the codon is adapted **(A)**. **(B)** The vaccine construct cloned into the vector pet28a(+) with the restriction sites Eco53kI and ScaI employed is shown in the red portion. Above is also the translated sequence for the red region.

The predicted MEV was ultimately subjected to in *silico cloning*, as it was predicted to be the most promising candidate for evoking immune responses and preventing *A. hydrophila* infection in individuals. To facilitate cloning, the vaccine construct was optimized using a codon adaptation tool tailored for the *E. coli K-12* expression system (59). Results indicated that approximately 95% of the codons were successfully modified to align with the expression system’s codon usage, suggesting that the MEV would be efficiently expressed in *E. coli* (**Figure 5A**). Codon optimization is crucial because the expression efficiency of codons varies among organisms, depending on their specific codon usage patterns (78). The optimized vaccine construct was then successfully cloned into a specialized vector, pet28a(+), with modified restriction sites as depicted in **Figure 5B**, respectively (79).

### Simulations of the docked complexes

**Figure 6:**
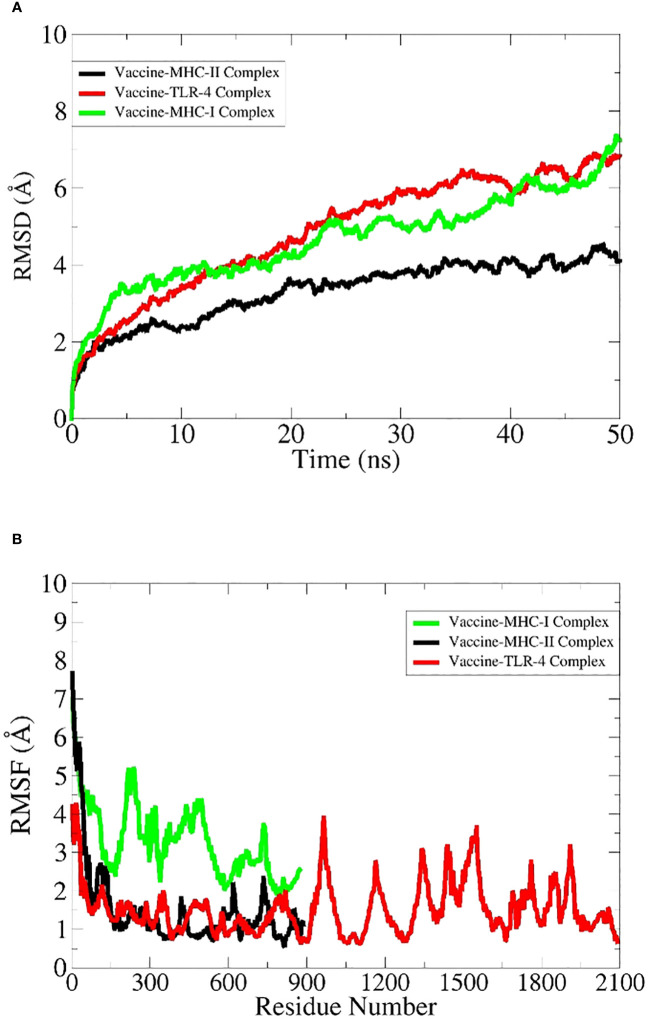
Vaccine-receptor molecular dynamics simulation analyses. **(A)** RMSD and **(B)** RMSF.

All docked complexes subjected to simulations yielded acceptable results. The root mean square deviation (RMSD) plot in **Figure 6A** showed linear deviations for each complex, indicating stability upon ligand binding and an absence of extreme deviations across the timeframe. This is a kind of ideal situation for our docked complexes. Root mean square fluctuation (RMSF) results in **Figure 6B** further clarify that amino acids within the active sites exhibit effective fluctuations conducive to efficient engagement and binding with their respective ligands. Molecular Mechanics Generalized Born Surface Area (MM-GBSA) and Molecular Mechanics Poisson–Boltzmann Surface Area (MM-PBSA) analyses demonstrated the stability of each complex upon binding (**Table 8**). Notably, the obtained energy values were significantly low, signifying maximal stability and efficient binding potentials of the MEV with the immune receptors.

**Table 8:** Intermolecular binding energies estimated by MMPGBSA/MMGBSA methods.

Parameter	Vaccine-TLR-4 complex (kcal/mol)	Vaccine-MHC-I complex (kcal/mol)	Vaccine-MHC-II complex (kcal/mol)
MMGBSA			
Van der Waals energy term	−218.24	−157.01	−166.29
Electrostatic energy term	−78.19	−55.06	−22.34
Gas phase energy term	−296.43	−212.07	−188.63
Solvation energy term	25.97	18.17	10.54
Net energy term	−270.46	−193.9	−178.09
MMPBSA			
Van der Waals energy term	−218.24	−157.01	−166.29
Electrostatic energy term	−78.19	−55.06	−22.34
Gas phase energy term	−296.43	−212.07	−188.63
Solvation energy term	30.29	21.57	11.59
Net energy term	−266.14	−190.5	−177.04

## Discussion

*A. hydrophila* dwells in freshwater and enrages people with its toxic substance, aerolysin (80). This bacterium casts a wide net of illnesses, ranging from sepsis and meningitis to bloody diarrhea and necrotizing wounds (81). Its lethality increases in susceptible groups, taking lives with a terrifying 50% mortality rate in conditions such as septicemia, leaving glaring reminders of its formidable menace (82). To stop this aquatic threat from affecting human health, prompt medical attention, careful water cleaning, and effective vaccine production techniques are required.

The main aim of this study was to design a MEV against *A. hydrophila* based on its virulence factor (aerolysin toxin) because virulence factors are important proteins associated with bacteria that help it induce and promote serious fatal infections in living organisms (83). Without essential factors, bacteria can be weak or no longer pathogenic (84). Numerous researchers have created MEVs against a wide range of diseases caused by bacteria while taking into account their virulence factors. In a recent work, for instance, a MEV construct was created using *Staphylococcus aureus* superantigens (85). Another study focused on designing a MEV against *Helicobacter pylori* (86). Additionally, MEV constructions targeting coronavirus spike proteins have been developed, with encouraging outcomes (38). Designed MEVs not only show robust immune responses virtually, but many studies have proved that these MEVs also induce robust immune responses in *in vivo* models. According to a study by Ramirez-Salinas et al. (31), the MEV construct against influenza A demonstrated a robust generation of neutralizing antibodies in mouse models. Additionally, Agallou et al. (28) presented a study in which BALB mice receiving a MEV construct made to target *Leishmania infantum* proteins produced a large number of interferons and interleukins.

As mentioned, aerolysin is among the most important virulence factors of *A. hydrophila* that actively contributes to the active killing of target cells in the human body (13). It is also a highly conserved gene present in different strains of *A. hydrophila* and other strains of the *Aeromonas* family. Osman et al. (87) isolated 17 strains of *A. hydrophila*, where three strains consist of the aerolysin gene upon analysis. Baloda et al. (88) conducted a research study where 89 strains of *A. hydrophila* and *A. sobria* were declared positive for the presence of an aerolysin gene upon PCR analysis. The construction of aerolysin-based MEVs against *A. hydrophila* will evoke the immune system of individuals when introduced into their bodies and will protect them from the effects of this deadly toxin. The sequence of aerolysin was extracted from UniProt, and structure was confirmed from PDB. Aerolysin was subjected to homology check by aligning its sequence with the human and normal flora bacteria proteome, which resulted in no significant match, which clarifies that there will be no occurring of autoimmune diseases due to the use of the designed vaccine. Alsubaiyel and Bukhari (89) used the same concept while designing MEVs against *Chlamydia psittaci.* Furthermore, a conservancy analysis of the aerolysin gene across all strains of *Aeromonas* species revealed that the majority of strains within the *Aeromonas* family possess the exact same aerolysin gene. This finding enhances the significance and diversity of our study, as the development of a MEV based on aerolysin would potentially provide coverage against almost all pathogenic strains of *A. hydrophila* and other pathogenic *Aeromonas* species.

The goal of the highly specialized, active acquired immune responses is to eradicate or stop the growth of infections (90). Memory B cells produced by adaptive immunity recognize the organism on future contacts following the initial recognition. Adaptive immunity’s immunological memory serves as the foundation for vaccination (91). Within the adaptive immune system, the main job of B- and T-lymphocyte cells is to create antibody-dependent cellular immunity against foreign invaders (92). The aerolysin sequence was utilized to identify the MHC-I, MHC-II, and B-cell epitopes. Aerolysin was found to have 14 B-cell epitopes of variable lengths, which were then processed to predict MHC-I and MHC-II epitopes. Epitopes with the lowest percentile scores were prioritized and selected because such epitopes ensure maximum binding with the immune receptors (93).

A total of 71 and 54 epitopes were predicted for MHC-I and MHC-II receptors, respectively. Each epitope underwent rigorous screening for allergenicity, solubility, toxicity, and antigenicity, resulting in 20 and 10 final candidates for MHC-II and MHC-I, respectively. These chosen epitopes were then joined via linkers to enhance the MEV’s stability and prevent self-binding. Linkers are used in multiple studies of MEV design because they provide integrity and effectivity to the vaccine candidate (94–96) To strengthen immune activation, cholera toxin B, an adjuvant, was also incorporated. Raheem et al. (97) also used cholera toxin B as an adjuvant in designing a MEV against *Vibrio cholera.* The final MEV consists of 666 amino acids and a molecular weight of 68,266.77 kDa. Its impressive stability is reflected in the low instability index value of 31.79 (98). Additionally, with a theoretical PI of 5.60 and a slightly higher abundance of negative residues, the vaccine candidate exhibits an acidic character. The hydrophilic character of the protein was indicated by its negative GRAVY score of −0.815, indicating that it functions normally within the body (99). According to antigenic studies, the vaccine has a half-life in the body of more than 20 h, which is typical for maximal proteins and indicates that it is highly antigenic.

As mentioned, the structure of MEV was predicted and refined to make it stable and suitable for docking. The refined structure was evaluated for stability through ERRAT and Ramachandran plots, which resulted in promising outcomes. The Ramachandran plot is divided into four regions, where each region explains a specific level of stability based on the arrangement of phi (angles between alpha carbon and nitrogen) and psi (angles between alpha carbon and carboxyl carbon) angles of each residue of MEV. Exploring each region: the most preferred area, or red region, is made up of amino acids with angle values (phi and psi) that do not exhibit steric hindrance, meaning that their molecules do not hinder one another (100). More amino acids in favored regions imply improved stability and docking flexibility (48). Secondly, in addition to the allowed regions, the dark brown zone also represents the flexibility of proteins. High processing difficulties are indicated by the yellow, or generously allowed region, which strongly impedes phi and psi angles. Steric hindrance severely limits the rotation of Phi and Psi angles in the pale yellow, or forbidden zone. The modeled MEV in the present study shows the maximum amino acids present in allowed regions, giving a combined value of 97.6% that shows the maximum stability of MEV.

In order to elicit a strong immunological response, the MEV must bind as much as possible to key immune cell receptors. MEV was paired with TLR4, MHC-I, and MHC-II, respectively. The maximum number of amino acids were implicated in the binding of both proteins, and the binding energies were more significant. Vaccine-TRL4-docked complex analysis revealed a binding energy of 1,023.4 kcal, 124 nonbonded contacts, 11 hydrogen bonds, and three disulfide bonds. There were a total of 28 residues implicated in these interactions. The binding energy of the docked complex of vaccine-MHC I was −923.2 kcal, and the number of bonded and nonbonded contacts was 289, with four hydrogen bonds, one disulfide bond, and four residues involved. The Vaccine-MHC II-docked complex revealed 110 residues implicated, four hydrogen bonds, and 200 nonbonded interactions.

Simulation studies for each complex were performed using AMBER. These are only performed to virtually check the behavior of complexes inside the body (101). A virtual environment is provided that is almost similar to the cellular environment, where the stability and performance of the complex are evaluated. This is a highly acceptable approach and can give researchers a clue to proceed with experimental applications (96). Amber is most widely used to simulate protein complexes effectively. The RMSD plot is widely used to check the overall stability of the protein complex with respect to a reference (102). It shows the combined deviations of residues in the protein complex, and extreme deviations in the structure demonstrate the instability of the complex. In our case, RMSD obtained for each complex showed acceptable residue deviations, which concludes that all the MEV–immune receptor complexes are stable and can perform well inside the body. Secondly, the results were supported by measuring energy values via the MM-GBSA and MM-PBSA approaches (57). The negative values of Vander Val forces and electrostatic energy conclude that the docked complexes have maximum interactions between their neutral and charged residues and have stable bindings. The energy of the gas phase showed high negative values for each complex, while the energy of solvation showed minute positive values that conclude that in the vacuumed phase, i.e., without a solvent environment, the complex has high stability and maximum bonding interactions, and upon exposure to a solvent environment, a minute input energy (energy of solvation) is required to adjust the complex to the introduced new environment. The overall net energy of each complex in both the MM-GBSA and MM-PBSA approaches has significant negative values in **Table 8**, which concludes that all the MEV–immune complexes have stable and maximum interactions in the cellular environment. Lastly, an RMSF plot was generated for the residues involved in the active site of the complexes, which demonstrates that each residue showed maximum fluctuations and concludes that all are actively involved in bindings and interactions (102).

As an exceptional MEV that satisfies all prerequisites, *in silico* cloning was guaranteed to virtually generate this MEV and make it accessible to the human race. In order to achieve this, we used the principles of recombinant DNA technology, which involved selecting an appropriate vector in order to insert the MEV sequence and re-express it using an expression system. Because *E. coli* has the highest rate of multiplication, it was chosen as the expression system, and the pet28a(+) vector was employed. The exponential increase of genomic data is a major factor in the rapid development of computational vaccine-designing strategies (103). Such analyses can direct the development of a safe vaccine against a variety of microbial pathogens and are extremely specific and efficient. All the requirements for a good vaccine were satisfied by the current vaccine design.

## Conclusion

This study concludes that the lack of availability of potential vaccines against *A. hydrophila* has created an alarming situation across humanity, and the introduction of potential vaccines can hinder the onset and progression of *A. hydrophila*-related infections. We produced a MEV candidate based on the aerolysin toxin (virulence factor) of *A. hydrophila* by processing it through different bioinformatics tools, and we were assured that this vaccine would be effective against this bacterium.

## Data availability statement

The original contributions presented in the study are included in the article/**Supplementary Material**. Further inquiries can be directed to the corresponding author.

## Author contributions

AA: Writing – original draft. MA: Writing – review & editing.
