# Supplementary material for: Construction of an aerolysin-based multi-epitope vaccine against Aeromonas hydrophila: an in silico machine learning and artificial intelligence-supported approach
Source: Front Immunol. 2024 Mar 1;15:1369890. doi: 10.3389/fimmu.2024.1369890 (PMC10940347; doi:10.3389/fimmu.2024.1369890)
Supplement: Supplementary file 1 [file Table_1.docx]

**Supplementary Table 1:** Table showed known strains of *Aeromonas* family that showed maximum sequence alignment with aerolysin (virulence factor). This simply illustrate the highly conserved nature of aerolysin gene among the species of its concerned family respectively.

| ***Aeromonas spp.*** | **No. of Organisms** | **Alignment %** |
| --- | --- | --- |
| [*Aeromonas sp. CA23*](https://www.ncbi.nlm.nih.gov/Taxonomy/Browser/wwwtax.cgi?id=2033032) | 1 | 100 |
| [*Aeromonas hydrophila*](https://www.ncbi.nlm.nih.gov/Taxonomy/Browser/wwwtax.cgi?id=644) | 8 | 97.36—95.94 |
| [*Aeromonas bestiarum*](https://www.ncbi.nlm.nih.gov/Taxonomy/Browser/wwwtax.cgi?id=105751) | 1 | 99.80—99.39 |
| [*Aeromonas sp. MR7*](https://www.ncbi.nlm.nih.gov/Taxonomy/Browser/wwwtax.cgi?id=2923419) | 1 | 99.59 |
| [*Aeromonas*](https://www.ncbi.nlm.nih.gov/Taxonomy/Browser/wwwtax.cgi?id=642) | 27 | 97.57—95.74 |
| [*Aeromonas hydrophila subsp. hydrophila ATCC 7966*](https://www.ncbi.nlm.nih.gov/Taxonomy/Browser/wwwtax.cgi?id=380703) | 1 | 98 |
| [*Aeromonas piscicola*](https://www.ncbi.nlm.nih.gov/Taxonomy/Browser/wwwtax.cgi?id=600645) | 1 | 97.36—97.16 |
| [*Aeromonas sp.*](https://www.ncbi.nlm.nih.gov/Taxonomy/Browser/wwwtax.cgi?id=647) | 1 | 98.98—97.99 |
| [*Aeromonas caviae*](https://www.ncbi.nlm.nih.gov/Taxonomy/Browser/wwwtax.cgi?id=648) | 1 | 97.16 |
| [*Aeromonas hydrophila subsp. hydrophila*](https://www.ncbi.nlm.nih.gov/Taxonomy/Browser/wwwtax.cgi?id=196023) | 3 | 96.55 |
| [*Aeromonas hydrophila AD9*](https://www.ncbi.nlm.nih.gov/Taxonomy/Browser/wwwtax.cgi?id=1453502) | 1 | 97 |
| [*Aeromonas hydrophila J-1*](https://www.ncbi.nlm.nih.gov/Taxonomy/Browser/wwwtax.cgi?id=1419584) | 1 | 97.5 |
| [*Aeromonas hydrophila NJ-35*](https://www.ncbi.nlm.nih.gov/Taxonomy/Browser/wwwtax.cgi?id=1416915) | 1 | 97 |
| [*Aeromonas sp. ASNIH4*](https://www.ncbi.nlm.nih.gov/Taxonomy/Browser/wwwtax.cgi?id=1636609) | 1 | 97 |
| [*Aeromonas sp. ASNIH6*](https://www.ncbi.nlm.nih.gov/Taxonomy/Browser/wwwtax.cgi?id=1758188) | 1 | 97 |
| [*Aeromonas sp. V90_14*](https://www.ncbi.nlm.nih.gov/Taxonomy/Browser/wwwtax.cgi?id=3044241) | 1 | 96.96 |
| [*Aeromonas hydrophila subsp. hydrophila AL09-71*](https://www.ncbi.nlm.nih.gov/Taxonomy/Browser/wwwtax.cgi?id=1321367) | 1 | 97.2 |
| [*Aeromonas hydrophila pc104A*](https://www.ncbi.nlm.nih.gov/Taxonomy/Browser/wwwtax.cgi?id=1418107) | 1 | 97.2 |
| [*Aeromonas sp. S41-2*](https://www.ncbi.nlm.nih.gov/Taxonomy/Browser/wwwtax.cgi?id=2990502) | 1 | 96.96 |
| [*Aeromonas sp. Y318-1*](https://www.ncbi.nlm.nih.gov/Taxonomy/Browser/wwwtax.cgi?id=2990508) | 1 | 96.55 |
| [*Aeromonas sp. 5HA1*](https://www.ncbi.nlm.nih.gov/Taxonomy/Browser/wwwtax.cgi?id=2699197) | 1 | 96.35 |
| [*Aeromonas sp. 2HA2*](https://www.ncbi.nlm.nih.gov/Taxonomy/Browser/wwwtax.cgi?id=2699194) | 1 | 96 |
| [*Aeromonas salmonicida*](https://www.ncbi.nlm.nih.gov/Taxonomy/Browser/wwwtax.cgi?id=645) | 2 | 96.15 |
| [*Aeromonas salmonicida subsp. pectinolytica 34mel*](https://www.ncbi.nlm.nih.gov/Taxonomy/Browser/wwwtax.cgi?id=1324960) | 1 | 96 |
| [*Aeromonas sp. DNRA1*](https://www.ncbi.nlm.nih.gov/Taxonomy/Browser/wwwtax.cgi?id=2729335) | 1 | 95.74 |
| [*Aeromonas sp. D3*](https://www.ncbi.nlm.nih.gov/Taxonomy/Browser/wwwtax.cgi?id=2990474) | 1 | 96.15 |
| [*Aeromonas dhakensis*](https://www.ncbi.nlm.nih.gov/Taxonomy/Browser/wwwtax.cgi?id=196024) | 1 | 96.2 |
| **Total** | **63** |  |
